# The relationship between community social risk factors and regional hospital-reported cash, negotiated, and chargemaster prices for 14 common services

**DOI:** 10.1186/s12913-024-10762-1

**Published:** 2024-03-06

**Authors:** Eva Chang, Wayne Psek

**Affiliations:** 1 Advocate Aurora Research Institute, Advocate Health, 945 N. 12th St, 53233 Milwaukee, WI USA; 2 Department of Health Policy and Management, Milken Institute School of Public Health, The George Washington University, Washington, DC USA

**Keywords:** Price transparency, Social risk factors, Hospital prices, Cash prices, Chargemaster prices

## Abstract

**Background:**

Social risk factors are key drivers of the geographic variation in spending in the United States but little is known how community-level social risk factors are associated with hospital prices. Our objective was to describe the relationship between regional hospital-reported prices and social risk factors by price type (chargemaster, cash, commercial, Medicare, and Medicaid).

**Methods:**

This cross-sectional analysis used newly available hospital-reported prices from acute general hospitals in 2022. The prices were for 14 common services. Prices were winsorized at 98%, wage index-adjusted, standardized by service, and aggregated to hospital service areas (HSAs). For social risk, we used 23 measures across 5 domains of social risk (socioeconomic position; race, ethnicity, and culture; gender; social relationships; and residential and community context). Spearman’s correlation was used to estimate associations between median prices and social risk by price type.

**Results:**

Prices were reported from 2,386 acute general hospitals in 45% (1,502 of 3,436) HSAs. Correlations between regional prices and other social risk factors varied by price type (range: -0.19 to 0.31). Chargemaster and cash prices were significantly correlated with the most community characteristics (10 of 23, 43%) followed by commercial prices (8, 35%). Medicare and Medicaid prices were only significantly correlated with 1 measure (all *p* < 0.01). All price types were significantly correlated with the percentage of uninsured (all *p* < 0.01). Chargemaster, cash, and commercial prices were positively correlated with percentage of Hispanic residents, residents with limited English proficiency, and non-citizens (all *p* < 0.05).

**Conclusions:**

While regional correlations between prices and social risk factors were weak across all prices, chargemaster, cash, and commercial prices were more like closely aligned with community-level social risk factors than the two public payers (Medicare and Medicaid). Chargemaster, cash, and commercial hospital prices appeared to be higher in socially disadvantaged communities. Further research is needed to clarify the relationship between prices and community social risk factors.

**Supplementary Information:**

The online version contains supplementary material available at 10.1186/s12913-024-10762-1.

## Background

Social risk factors have become increasingly important in the national discourse on healthcare payment in the United States. Payers and quality-related organizations are increasingly accounting for social risk factors in healthcare quality benchmarks, value-based payments, and accreditation standards, while health systems and individual providers are rapidly expanding inclusion of patients’ social risk factors into clinical and community care [1–4]. Defined as a “set of constructs that capture key ways in which social processes and social relationship could influence key health-related outcomes [1],” social risk factors have been identified as key drivers of geographic variation in healthcare utilization and spending [5–7]. While evidence documenting the inclusion and use of social risk factors in clinical care is growing, little is known about how social risk factors relate to administrative practices such as service prices, especially as prices may influence access to and equity in clinical services [8–11].

Hospital prices are a major factor underlying the growth in hospital expenditures, which represent almost 6% of US GDP at over $1.11 trillion [12]. Since January 2021, under the Hospital Price Transparency Rule (45 CFR Part 180), the Center for Medicare & Medicaid Services (CMS) has required hospitals to report standard charges for at least 300 services that can be scheduled in advance (“shoppable services”) [13, 14]. The Rule requires that hospitals report gross charges (or chargemaster price), cash prices, and payer-specific negotiated prices. Chargemaster prices are also known as the full or list price that providers charge for their services, without any discounts. Payer-specific negotiated prices are the prices that a hospital negotiated with a third party payer or commercial insurance plan; government insurers, including insurance for elderly (Medicare) and low income (Medicaid) individuals, generally pay much lower prices that commercial insurers [15]. Finally, of particular interest are cash prices, or the allowed maximum amount that self-pay patients will be billed for hospital services. Many patients who are billed cash prices are part of the estimated 32% of working adults who are uninsured or underinsured in 2022 [16]. While cash prices have been understudied due to lack of data availability, recent studies about cash prices have been found them to be highly variable across hospitals and approximately 60% higher than payer-negotiated prices [17]. Further, cash prices for ED visits have been found to be lower in counties with high poverty levels and higher among for-profit hospitals and larger hospitals [18].

Since high hospital prices are likely to hinder access to healthcare services within socially disadvantaged communities (i.e., those with a high proportion of patients with socially disadvantaged conditions) and impose financial burdens on uninsured patients and privately insured patients using out-of-network services [8–10, 19], it is important to understand the relationship between hospital prices and the social conditions of the communities that hospitals serve. Community-level social risk factors describe the social conditions of neighborhoods such as housing quality and access to transportation [20]. These measures are important tools for policymakers and healthcare providers for identifying high need areas, tracking changes in communities, and designing interventions that target these needs [20, 21]. Therefore, we correlated publicly available measures of social risk with hospital-reported price data for 14 hospital services to describe community context around different hospital price types (chargemaster, cash, commercial, Medicare, and Medicaid). Findings from this study may inform U.S. policy efforts tracking healthcare affordability to ensuring more equitable access to care for disadvantaged patients.

## Methods

### Study data and population

This cross-sectional analysis of publicly available, secondary data used a limited dataset of hospital-reported prices (chargemaster, cash, negotiated commercial, Medicare, and, Medicaid) for 14 common services, collected and provided by Turquoise Health (https://turquoise.health/researchers) [22]. The dataset, downloaded in May 2022, contained all machine-readable price data reported and billed by U.S. hospitals, as required by the Hospital Price Transparency Rule [13]. Turquoise Health updates these data quarterly and does not retain historical information; prices analyzed were current as of May 9, 2022. We included short-term general medical and surgical hospitals that reported cash, commercial, Medicare, or Medicaid prices for 14 healthcare services. Eleven of the services were defined as shoppable services by the CMS; 3 were high-volume emergency department (ED) services. Data on social risk factors came from the U.S. Census (2010 Decennial Census [23] and 2015–2019 American Community Survey [24]) and the Robert Graham Center [25]. The unit of analysis was health service areas (HSAs), from the Dartmouth Atlas [26].

The study was deemed non-human subject research by the George Washington University Institutional Review Board since we only used publicly available data.

### Study variables

We assessed 5 different types of hospital-reported prices—chargemaster, cash, Medicare, Medicaid, and commercial—for the set of 14 hospital services. The 14 services included knee arthroscopic cartilage removal, uterine and adnexa procedures (nonmalignancy), 3 levels of new patient office or other outpatient visit, (30, 45, and 60 min), colonoscopy (diagnostic), magnetic resonance imaging scan of brain before and after contrast, computed tomography scan (pelvis with contrast), ultrasound of abdomen (complete), kidney function blood test panel, electrocardiogram (routine with interpretation and report), and 3 levels of emergency (levels 3, 4, and 5). Prices did not distinguish care settings. Prices were winsorized at 98% and adjusted with the 2022 CMS wage index to account for regional differences in costs [27, 28]. Service-specific median prices were calculated for each hospital and price type and then aggregated within HSAs, weighted by the number of hospital beds.

We identified measures of social risk using a conceptual framework developed by the National Academy of Sciences, Engineering, and Medicine (NASEM) [1]. The framework identifies 5 domains of social risk (socioeconomic position; race, ethnicity, and cultural context; gender; social relationships; and residential and community context) that are expected to effect healthcare use, healthcare outcomes, and resource use [1]. We identified publicly available indicators available at the zip code level, mapped the indicators to 1 of the 5 domains and tested HSA-level correlation between indicators [7]. Among highly correlated measures (*r* ≥ 0.7) within the same domain and construct, we chose the measure that was more closely aligned with poor access to healthcare. We selected 23 measures for this analysis. Appendix A includes definitions and sources for the included measures and Appendix B includes the HSA-level correlations between the measures.

### Statistical analysis

We calculated descriptive statistics of each service by type of price. We then performed pairwise Spearman’s correlations to estimate associations between median prices and social risk for regions with ≥ 8 services reported. We only included the regions that had hospitals reporting more than half of the services to reduce potential biases from regions with fewer services reported. We adjusted for multiple comparisons using the Bonferroni correction.

We conducted three sensitivity analyses to test the robustness of the findings. First, we included all regions with at least 1 service reported. Second, we calculated service-specific correlations to assess each services relationship with the social risk factors. Finally, we aggregated to the 306 health referral regions (HRRs) and repeated our analyses to test how sensitive our results were to the geographic unit.

All analyses were conducted using Stata version 17.0 (Stata Corporation, College Station, TX). Two-sided *p* < 0.05 was considered statistically significant.

## Results

### Variation in prices and reporting

Prices were reported from 2,386 acute general hospitals in 45% (1,502 of 3,436) HSAs. Most hospitals were not-for-profit (45%), non-urban (78%) and not affiliated with medical schools (64%). The number of HSAs with at least 1 hospital reporting the service varied by service and price type (Table 1). Generally, chargemaster, cash, and commercial prices were reported more frequently while Medicaid prices were reported least frequently. For example, 1,401 HSAs had at least 1 hospital reporting a chargemaster price for MRI scan of the brain compared to 764 HSAs reporting a Medicaid price. Median HSA-level prices also varied widely across services and price types (Table 1). Median chargemaster prices were the highest while Medicare and Medicaid prices were the lowest. On average, chargemaster prices were 4 to 5 times higher than Medicare and Medicaid prices though this ratio varied by service. Chargemaster prices were approximately 2.6 times higher than Medicare prices for 30-minute new patient visits and 18 times higher than Medicare prices for kidney function blood test panels. Finally, median cash prices were about 55% of chargemaster prices yet more than 2 times higher than Medicare prices.

**Table 1:** HSA-level summary statistics of hospital-reported prices, by service and price type, May 2022

Medical services	Chargemaster		Cash		Commercial		Medicare		Medicaid	
	N	Median (IQR)	N	Median (IQR)	N	Median (IQR)	N	Median (IQR)	N	Median (IQR)
Uterine and adnexa procedures, nonmalignancy	700	38,205 (28,175 − 54,423)	693	20,350 (13,047 − 31,528)	992	14,682 (10,652 − 19,761)	733	8,411 (7,670-9,516)	374	6,960 (5,380-9,490)
Knee arthroscopic cartilage removal	687	9,999 (5,420 − 18,964)	697	5,257 (2,668-9,868)	895	4,837 (3,014 − 7,705)	659	2,838 (2,634-2,976)	431	1,799 (963-2,837)
Colonoscopy	920	2,994 (1,971-4,998)	929	1,726 (980-3,022)	1,071	1,803 (1,116-2,752)	820	798 (744–845)	576	627 (431–974)
MRI scan of brain before and after contrast	1,401	4,533 (3,195-6,198)	1,349	2,556 (1,581-3,794)	1,301	1,749 (950-3,109)	1,003	392 (377–444)	764	520 (346–876)
CT scan, pelvis, with contrast	1,388	2,606 (1,798-3,637)	1,330	1,461 (940-2,131)	1,265	1,096 (542-1,764)	954	188 (182–207)	735	290 (188–439)
Ultrasound of abdomen, complete	1,423	1,093 (740-1,624)	1,368	626 (402–950)	1,317	467 (234–777)	1,023	115 (111–123)	786	132 (90–208)
Kidney function blood test panel	1,378	179 (94–301)	1,339	91 (49–159)	1,216	59 (19–130)	896	10 (9–11)	674	11 (9–22)
Electrocardiogram, routine, with interpretation and report	369	97 (55–223)	346	61 (28–131)	398	46 (28–158)	215	18 (16–52)	205	23 (16–44)
New patient office or other outpatient visit, typically 30 min	1,095	298 (214–418)	1,017	176 (115–269)	902	168 (122–239)	555	112 (84–124)	474	77 (59–123)
New patient office of other outpatient visit, typically 45 min	1,073	402 (281–567)	992	235 (150–360)	880	223 (154–313)	537	134 (117–160)	468	114 (78–147)
New patient office of other outpatient visit, typically 60 min	1,028	506 (355–713)	941	289 (183–458)	821	276 (193–392)	506	172 (119–211)	437	124 (80–172)
Emergency level 3	1,404	1,054 (665-1,524)	1,352	553 (350–883)	1,321	630 (381–974)	1,016	236 (223–251)	774	179 (117–266)
Emergency level 4	1,402	1,678 (1,045 − 2,358)	1,351	881 (550-1,378)	1,318	971 (591-1,481)	1,017	371 (352–404)	770	279 (163–412)
Emergency level 5	1,402	2,421 (1,515-3,500)	1,350	1,274 (798-2,014)	1,315	1,319 (806-2,051)	1,014	532 (497–581)	764	364 (218–593)
Aggregate medical services	1,482	1,399 (900-2,196)	1,433	769 (486-1,310)	1,418	847 (502-1,346)	1,176	335 (212–414)	901	246 (145–407)

Abbreviations: CT = computed tomography; IQR = interquartile range; MRI = magnetic resonance imaging

### Correlations between prices and social risk factors

Among HSAs with prices for ≥ 8 services, chargemaster and cash prices were correlated with the most social risk factors (10 of 23, 43%) followed by commercial (8, 35%). Notably, Medicare and Medicaid were both correlated with only 1 of the included measure (4%). Table 2 presents the number of social risk factors and their domains that were significantly correlated each price type.

**Table 2:** Number (percent) of social risk factors correlated to hospital-reported price, by price type

Price type	N (of 3436)	Total measures (*n* = 23)	Social Risk Domains				
			Socioeconomic position (*n* = 7)	Race, ethnicity, and community context (*n* = 5)	Gender (*n* = 1)	Social relationships (*n* = 3)	Residential and community context (*n* = 7)
Chargemaster	1285	10 (43%)	2 (29%)	4 (80%)	0 (0%)	1 (33%)	3 (43%)
Cash	1231	10 (43%)	3 (43%)	3 (60%)	0 (0%)	1 (33%)	3 (43%)
Commercial	1225	8 (35%)	2 (29%)	3 (60%)	0 (0%)	1 (33%)	2 (29%)
Medicare	915	1 (4%)	1 (14%)	0 (0%)	0 (0%)	0 (0%)	0 (0%)
Medicaid	652	1 (4%)	1 (14%)	0 (0%)	0 (0%)	0 (0%)	0 (0%)

Note: Correlations are among HSAs with prices for ≥ 8 services reported

**Fig. 1:**
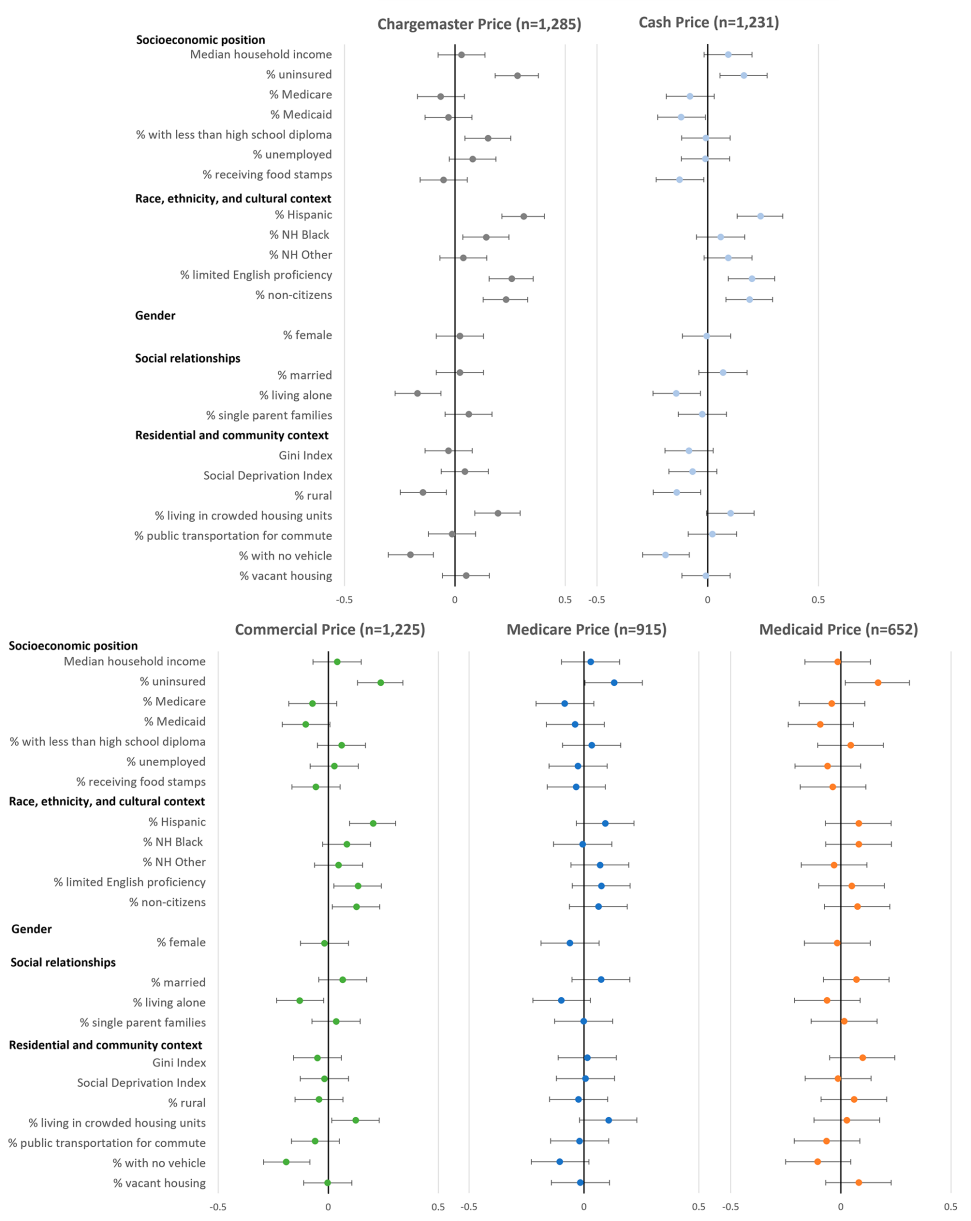
Spearman correlations between median hospital-reported prices and social risk factors by price type Note: Correlations (r_s_) are among HSAs with prices for ≥ 8 services reported and include Bonferroni-adjusted 95% CIs.

Figure 1 presents the correlations between median prices and social risk factors by price type among HSAs with prices for ≥ 8 services. (Spearman correlations and Bonferroni-adjusted 95% CIs are provided in Appendix C.) Correlations were weak, ranging from − 0.19 to 0.31. All price types were significantly and positively correlated with a measure of socioeconomic position, percentage of the population that was uninsured (range: 0.13 to 0.28, all *p* < 0.01) (i.e., prices were higher among HSAs with more uninsured residents). While no other measures were correlated with Medicare or Medicaid prices, common measures in each of the other domains, except gender, were found to be significantly correlated with chargemaster, cash, and commercial prices. Correlations were generally consistent in direction across price types. Most notably, within the race, ethnicity, and culture domain, 3 of 5 included measures (percentage of the population that was Hispanic, had limited English proficiency, and were noncitizens) were significantly, positively correlated with chargemaster, cash, and commercial prices; these measures were highly correlated with each other (Appendix B). Within the social relationships domain, the percentage of the population that lived alone was negatively correlated with chargemaster, cash, and commercial prices (all *p* < 0.01). Two of 8 included measures within the residential and community context domain were also correlated with the same 3 price types; percentage of the population living in crowded housing units was positively correlated with prices while the percentage with no vehicle was negatively correlated with prices (all *p* < 0.05).

In contrast, many of the included measures within socioeconomic position, social relationships, and residential and community context were not significantly correlated with any of the price types. These included median household income, percentage of the population with Medicare, unemployed, married, in single parent families, using public transportation to commute, and vacant housing. Prices were also not significantly correlated with gender or the composite community indices (Social Deprivation Index and Gini Index).

### Sensitivity analyses

We tested the sensitivity of our findings by including all HSAs that had at least 1 service reported and by aggregated to the HRR-level. Though statistical significance varied, the correlations were generally consistent in direction; these results are presented in Appendix D. We also calculated service-specific correlations with the social risk factors and found wide variation in the significance and direction of the correlations by service (available upon request).

## Discussion

This cross-sectional study examined a comprehensive set of social risk factors to describe the regional variation in community-level social risk factors and chargemaster, cash, commercial, Medicare, and Medicaid hospital prices. We found that while regional correlations between prices and social risk factors were weak across all prices, chargemaster, cash, and commercial prices were more closely aligned with social risk factors than the two public payers (Medicare and Medicaid). All pricing categories were correlated to a measure of socioeconomic position (percentage uninsured). Chargemaster, cash, and commercial prices were correlated with one or more included measures of social risk from each domain except gender, while Medicaid and Medicare prices were only correlated with percentage uninsured.

To our knowledge, this is the first study to examine the correlation between regional hospital-reported prices and a comprehensive set of social risk factors. This is consistent with studies that focus on social risk factors and spending (which is a function of both price and volume). Two previous studies examining social risk factors and geographic variation in spending also found significant, positive relationship between spending and percentage uninsured. Specifically, in addition to percentage uninsured being positively correlated with HRR-level spending for private, Medicare, and Medicaid payers, Cooper et al. also found relationships with other measures of socioeconomic position (mean household income, percentage with bachelor’s degree, employment rate, percentage in poverty) to not be significantly different from zero [5]. Zhang et al. found multiple measures across different domains (median household income, percentage uninsured, percentage noncitizens, social associations, and percentage with severe housing problems) to be associated with county-level Medicare spending [7]. While more work is needed to verify our early, descriptive findings, these studies support our findings and highlight the need to better understand how different social risk factors influence geographic variation in hospital pricing and may inform policy and provider prioritization of community health needs.

Our study is cross-sectional, so we cannot assess if there is a causal relationship between hospital prices and social risk factors. However, the statistically significant correlations between several regional social risk factors and chargemaster, cash, and commercial hospital prices suggest that hospitals may be sensitive to the social conditions around their hospital. For example, higher prices in areas with more uninsured residents may suggest that hospitals in these regions have increased prices to account for greater levels of uncompensated care [29, 30]. Additionally, the lack of significant correlations between community-level social risk factors and Medicare and Medicaid prices suggests that since Medicare and Medicaid reimbursement are set at national and state levels, these prices may be less sensitive to community-level factors. Further research is needed to clarify these relationships.

Notably, we also observed that regional chargemaster, cash, and commercial hospital prices were significantly and positively correlated with several measures included in the race, ethnicity, and cultural context domain including percentage of Hispanic residents, residents with limited English proficiency, and non-citizens. Prior studies have similarly found higher Medicare charges for ED services among hospitals serving greater Hispanic and African American patient populations [29]. These findings suggest that hospital prices may be higher in communities of residents who may have more difficulty navigating health services and less likely to negotiate or dispute prices [31–33]. However, higher hospital prices for services may also reflect several different factors, including payer mix, hospital characteristics, hospital expenses and market competition [34–36]. While these higher prices may also be due efforts to recoup uncompensated care, higher hospital prices may also reflect pricing discrimination efforts such as raising prices for privately insured patients and decreasing acceptance of Medicaid [11]. More research into hospital pricing practices is needed to ensure equitable access to hospital services.

### Limitations

This study has several important limitations. A key limitation is that data completeness and quality are contingent on hospital reporting [18, 37–40] and more than half of the HSAs were excluded in any of our assessments. However, we chose not to focus on HSAs with less than half (i.e., 8) of the services reported because those with fewer reported services were more likely to be skewed by extreme prices. Prices for services that are less frequently reported (e.g., electrocardiogram) would be less likely to be included in the aggregate set of services. Further, prior research found compliance with reporting varies by several factors including hospital size, IT preparedness, and financial resources, so our sample is more likely to include these hospitals [37, 41]. Additionally, while some of the variation in reported prices may be due to different hospital costs and profit margins, a portion of the variation is likely due to differences in what hospitals include in the price for the same service. Also, the reported price does not reflect the true cost of a service since it does not include the professional fees or patient cost-sharing information. Further, price type was largely classified based on name of insurance plan so several insurance plans may have been miscategorized. However, our calculated median Medicare price for each service was comparable to the prices reported in Medicare.gov’s Procedure Price Lookup tool. This study also used a limited set of 14 services; the inclusion of more services may change the relationships with social risk factors. Finally, the data used are cross-sectional and limits our ability to establish causal relationships between prices and social risk factors. As more data are released, future studies will be better able to assess whether community-level social risk effects hospital prices.

## Conclusion

Using national data on hospital prices, our study assessed the regional variation in social risk factors for chargemaster, cash, commercial, Medicare, and Medicaid hospital prices in the US. In addition to identifying several measures of social risk correlated with hospital prices, we found considerable variation in hospital prices and correlation between social risk factors and price to be low. However, chargemaster, cash, and commercial hospital prices appeared to be higher in socially disadvantaged communities. More research is needed to understand why communities of higher social risk were correlated with higher prices for hospital services and how prices may impact access to hospital services in these communities.

### Electronic supplementary material

Below is the link to the electronic supplementary material.

Supplementary Material 1

## Data Availability

The datasets analyzed during the current study are available from the corresponding author upon reasonable request.

## Abbreviations

CMS: Center for Medicare & Medicaid Services
ED: Emergency department
HRRs: Health referral regions
HSAs: Health service areas
NASEM: National Academy of Sciences, Engineering, and Medicine
