# Supplementary material for: The relationship between community social risk factors and regional hospital-reported cash, negotiated, and chargemaster prices for 14 common services
Source: BMC Health Serv Res. 2024 Mar 6;24:299. doi: 10.1186/s12913-024-10762-1 (PMC10918866; doi:10.1186/s12913-024-10762-1)
Supplement: Supplementary file 1 — Supplementary Material 1 [file 12913_2024_10762_MOESM1_ESM.docx]

# Appendix A. Definitions and sources of all considered social risk factors

| **Domain/ construct** | **Measure** | **Definition** | **Source** |
| --- | --- | --- | --- |
| **Socioeconomic position** | | | |
| Income | **Median household income** | Median household income (2019 inflation adjusted dollars) | ACS 5-Year Data (2015-2019) (24) |
|  | % in poverty | Percent families and people whose income in past 12 months is below poverty level | ACS 5-Year Data (2015-2019) (24) |
| Insurance | **% uninsured** | Percent of civilian noninstitutionalized population with no health insurance | ACS 5-Year Data (2015-2019) (24) |
|  | **% Medicare** | Percent of civilian noninstitutionalized population with Medicare coverage alone or in combination | ACS 5-Year Data (2015-2019) (24) |
|  | **% Medicaid** | Percent of civilian noninstitutionalized population with Medicaid/means-tested public coverage alone | ACS 5-Year Data (2015-2019) (24) |
|  | % private/other | Percent of civilian noninstitutionalized population with private or other health insurance | ACS 5-Year Data (2015-2019) (24) |
| Education | **% with less than high school diploma** | Percent of population 25 years and older with less than a high school diploma | ACS 5-Year Data (2015-2019) (24) |
|  | % with college degree or higher | Percent of population 25 years and older with a Bachelor's degree or higher | ACS 5-Year Data (2015-2019) (24) |
| Occupation | **% unemployed** | Percent of population 16 years and over in the civilian labor force who are unemployed | ACS 5-Year Data (2015-2019) (24) |
| Access to economic resources | **% receiving food stamps** | Percent of households with food stamp/SNAP benefits in the past 12 months (2019 inflation adjusted dollars) | ACS 5-Year Data (2015-2019) (24) |
| **Race, Ethnicity, and Community Context** | | | |
| Race and Ethnicity | **% Hispanic** | Percent of population who are Hispanic or Latino (of any race) | ACS 5-Year Data (2015-2019) (24) |
|  | **% NH Black** | Percent of population who are Black or African American alone (not Hispanic or Latino) | ACS 5-Year Data (2015-2019) (24) |
|  | **% NH Other** | Percent of population who are not Hispanic or Latino, White, or Black or African American | ACS 5-Year Data (2015-2019) (24) |
| Language | % speak a language other than English at home | Percent of population 5 years and older who speak a language other than English at home | ACS 5-Year Data (2015-2019) (24) |
|  | **% limited English proficiency** | Percent of population 5 years and older who speak English less than very well | ACS 5-Year Data (2015-2019) (24) |
| Nativity | % foreign born | Percent of population who were foreign born | ACS 5-Year Data (2015-2019) (24) |
|  | **% non-citizens** | Percent of population who were non-citizens | ACS 5-Year Data (2015-2019) (24) |
| **Gender** | | | |
| Gender | **% female** | Percent of population who were female | ACS 5-Year Data (2015-2019) (24) |
| **Social Relationships** | | | |
| Marital status | **% married** | Percent of population 15 years and older who now married, except separated | ACS 5-Year Data (2015-2019) (24) |
| Living alone | **% living alone** | Percent of households where householder is living alone | ACS 5-Year Data (2015-2019) (24) |
| Social support | **% single parent families** | Percent of households where there is a single householder with one or more people under 18 years | ACS 5-Year Data (2015-2019) (24) |
| **Residential and Community Context** | | | |
| Neighborhood deprivation | **Gini Index** | Gini Index | ACS 5-Year Data (2015-2019) (24) |
|  | **Social Deprivation Index** | Social Deprivation Index | Robert Graham Center (2015-2019)(25) |
|  | Community Deprivation Index | Community Deprivation Index | Community Deprivation Index (2018)(42) |
| Urbanicity and Rurality | **% rural** | Percent rural areas | Decennial Census (2010) |
| Housing | **% living in crowded housing units** | Percent of occupied housing units with 1.01 or more occupants per room | ACS 5-Year Data (2015-2019) (24) |
| Other Environmental Measures of Residential and Community Context | **% public transportation for commute** | Percent of workers 16 years and over who commute to work using public transportation (except taxicab) | ACS 5-Year Data (2015-2019) (24) |
|  | **% with no vehicle** | Percent of total occupied housing units with no vehicle available | ACS 5-Year Data (2015-2019) (24) |
|  | **% vacant housing** | Percent of housing units that are vacant | ACS 5-Year Data (2015-2019) (24) |

Abbreviations: ACS=American Community Survey

Included measures are bolded.

# Appendix B. HSA-level correlations between measures of social risk, by social risk domain

Table B-1. HSA-level correlations between measures of socioeconomic position

| Socioeconomic position measures | Median household income | % in poverty | % uninsured | % Medicare | % Medicaid | % private/other | % with less than high school diploma | % with college degree or higher | % unemployed | % receiving food stamps |
| --- | --- | --- | --- | --- | --- | --- | --- | --- | --- | --- |
| **Median household income** | 1.000 |  |  |  |  |  |  |  |  |  |
| % in poverty | **-0.817** | 1.000 |  |  |  |  |  |  |  |  |
| **% uninsured** | -0.436 | 0.483 | 1.000 |  |  |  |  |  |  |  |
| **% Medicare** | -0.497 | 0.188 | -0.028 | 1.000 |  |  |  |  |  |  |
| **% Medicaid** | -0.580 | **0.705** | 0.207 | 0.123 | 1.000 |  |  |  |  |  |
| % private/other | **0.782** | **-0.738** | -0.549 | -0.507 | **-0.753** | 1.000 |  |  |  |  |
| **% with less than high school diploma** | -0.573 | 0.663 | 0.587 | 0.038 | 0.635 | -0.673 | 1.000 |  |  |  |
| % with college degree or higher | **0.736** | -0.599 | -0.388 | -0.331 | -0.549 | 0.663 | -0.684 | 1.000 |  |  |
| **% unemployed** | -0.249 | 0.455 | 0.225 | -0.146 | 0.535 | -0.363 | 0.389 | -0.208 | 1.000 |  |
| **% receiving food stamps** | **-0.717** | **0.799** | 0.333 | 0.217 | **0.805** | **-0.736** | 0.650 | -0.625 | 0.545 | 1.000 |

Included measures are bolded.

Table B-2. HSA-level correlations between measures of race, ethnicity, and community context

| Race, ethnicity, and community context measures | % Hispanic | % NH Black | % NH Other | % speak a language other than English at home | % limited English proficiency | % foreign born | % non-citizens |
| --- | --- | --- | --- | --- | --- | --- | --- |
| **% Hispanic** | 1.000 |  |  |  |  |  |  |
| **% NH Black** | 0.186 | 1.000 |  |  |  |  |  |
| **% NH Other** | 0.378 | 0.120 | 1.000 |  |  |  |  |
| % speak a language other than English at home | **0.854** | 0.190 | 0.482 | 1.000 |  |  |  |
| **% limited English proficiency** | **0.842** | 0.239 | 0.423 | **0.949** | 1.000 |  |  |
| % foreign born | **0.823** | 0.262 | 0.497 | **0.889** | **0.892** | 1.000 |  |
| **% non-citizens** | **0.833** | 0.268 | 0.445 | **0.875** | **0.898** | **0.964** | 1.000 |

Included measures are bolded.

Table B-3. HSA-level correlations between measures of social relationships

| Social relationships measures | % married | % living alone | % single parent families |
| --- | --- | --- | --- |
| **% married** | 1.000 |  |  |
| **% living alone** | -0.254 | 1.000 |  |
| **% single parent families** | -0.598 | -0.124 | 1.000 |

Included measures are bolded.

Table B-4. HSA-level correlations between measures of residential and community context

| Residential and community context measures | Gini Index | Social Deprivation Index | Community Deprivation Index | % rural | % living in crowded housing units | % public transportation for commute | % with no vehicle | % vacant housing |
| --- | --- | --- | --- | --- | --- | --- | --- | --- |
| **Gini Index** | 1.000 |  |  |  |  |  |  |  |
| **Social Deprivation Index** | 0.460 | 1.000 |  |  |  |  |  |  |
| Community Deprivation Index | 0.433 | **0.823** | 1.000 |  |  |  |  |  |
| **% rural** | 0.021 | 0.032 | 0.344 | 1.000 |  |  |  |  |
| **% living in crowded housing units** | 0.119 | 0.487 | 0.321 | -0.210 | 1.000 |  |  |  |
| **% public transportation for commute** | -0.005 | -0.095 | -0.310 | -0.558 | 0.166 | 1.000 |  |  |
| **% with no vehicle** | 0.391 | 0.589 | 0.406 | -0.053 | 0.103 | 0.145 | 1.000 |  |
| **% vacant housing** | 0.294 | 0.222 | 0.544 | 0.628 | -0.081 | -0.402 | 0.115 | 1.000 |

Included measures are bolded.

# Appendix C. Spearman correlations and Bonferroni-adjusted 95% CIs between median hospital-reported prices and social risk factors by price type, among HSAs with prices for ≥8 services reported.

| **Social risk factors** | **Chargemaster Price (n=1,285)** | | **Cash Price (n=1,231)** | | **Commercial Price (n=1,225)** | | **Medicare Price (n=915)** | | **Medicaid Price (n=652)** | |
| --- | --- | --- | --- | --- | --- | --- | --- | --- | --- | --- |
|  | r_s_ | Bonferroni 95% CI | r_s_ | Bonferroni 95% CI | r_s_ | Bonferroni 95% CI | r_s_ | Bonferroni 95% CI | r_s_ | Bonferroni 95% CI |
| **Socioeconomic position** | | | | | | | | | | |
| Median household income | 0.030 | -0.08, 0.14 | 0.093 | -0.02, 0.20 | 0.040 | -0.07, 0.15 | 0.029 | -0.10, 0.15 | -0.014 | -0.16, 0.14 |
| % uninsured | 0.283*** | 0.18, 0.38 | 0.164*** | 0.06, 0.27 | 0.238*** | 0.13, 0.34 | 0.131** | 0.00, 0.25 | 0.169** | 0.02, 0.31 |
| % Medicare | -0.064 | -0.17, 0.04 | -0.080 | -0.19, 0.03 | -0.072 | -0.18, 0.04 | -0.084 | -0.21, 0.04 | -0.041 | -0.19, 0.11 |
| % Medicaid | -0.029 | -0.14, 0.08 | -0.119** | -0.23, -0.01 | -0.102* | -0.21, 0.01 | -0.038 | -0.16, 0.09 | -0.093 | -0.24, 0.06 |
| % with less than high school diploma | 0.151*** | 0.04, 0.25 | -0.008 | -0.12, 0.10 | 0.061 | -0.05, 0.17 | 0.034 | -0.09, 0.16 | 0.045 | -0.11, 0.19 |
| % unemployed | 0.081 | -0.03, 0.19 | -0.010 | -0.12, 0.10 | 0.028 | -0.08, 0.14 | -0.026 | -0.15, 0.10 | -0.061 | -0.21, 0.09 |
| % receiving food stamps | -0.051 | -0.16, 0.06 | -0.126** | -0.23, -0.02 | -0.057 | -0.16, 0.05 | -0.034 | -0.16, 0.09 | -0.036 | -0.18, 0.11 |
| **Race, Ethnicity, and Cultural Context** | | | | | | | | | | |
| % Hispanic | 0.313*** | 0.21, 0.41 | 0.239*** | 0.13, 0.34 | 0.203*** | 0.10, 0.31 | 0.093 | -0.03, 0.22 | 0.081 | -0.07, 0.23 |
| % NH Black | 0.142*** | 0.04, 0.24 | 0.059 | -0.05, 0.17 | 0.084 | -0.03, 0.19 | -0.006 | -0.13, 0.12 | 0.082 | -0.07, 0.23 |
| % NH Other | 0.039 | -0.07, 0.14 | 0.093 | -0.02, 0.20 | 0.047 | -0.06, 0.16 | 0.071 | -0.06, 0.20 | -0.031 | -0.18, 0.12 |
| % limited English proficiency | 0.258*** | 0.16, 0.35 | 0.200*** | 0.09, 0.30 | 0.135*** | 0.03, 0.24 | 0.075 | -0.05, 0.20 | 0.049 | -0.10, 0.20 |
| % non-citizens | 0.231*** | 0.13, 0.33 | 0.190*** | 0.08, 0.29 | 0.128** | 0.02, 0.23 | 0.063 | -0.06, 0.19 | 0.076 | -0.07, 0.22 |
| **Gender - % female** | 0.023 | -0.08, 0.13 | -0.005 | -0.11, 0.10 | -0.018 | -0.13, 0.09 | -0.062 | -0.19, 0.07 | -0.016 | -0.17, 0.13 |
| **Social Relationships** | | | | | | | | | | |
| % married | 0.023 | -0.08, 0.13 | 0.070 | -0.04, 0.18 | 0.066 | -0.04, 0.17 | 0.075 | -0.05, 0.20 | 0.072 | -0.08, 0.22 |
| % living alone | -0.169*** | -0.27, -0.06 | -0.141*** | -0.25, -0.03 | -0.130** | -0.24, -0.02 | -0.099 | -0.22, 0.03 | -0.063 | -0.21, 0.09 |
| % single parent families | 0.063 | -0.04, 0.17 | -0.024 | -0.13, 0.09 | 0.036 | -0.07, 0.14 | -0.002 | -0.13, 0.12 | 0.015 | -0.13, 0.16 |
| **Residential and Community Context** | | | | | | | | | | |
| Gini Index | -0.029 | -0.14, 0.08 | -0.085 | -0.19, 0.02 | -0.050 | -0.16, 0.06 | 0.014 | -0.11, 0.14 | 0.099 | -0.05, 0.25 |
| Social Deprivation Index | 0.046 | -0.06, 0.15 | -0.068 | -0.18, 0.04 | -0.018 | -0.13, 0.09 | 0.006 | -0.12, 0.13 | -0.012 | -0.16, 0.14 |
| % rural | -0.145*** | -0.25, -0.04 | -0.140*** | -0.25, -0.03 | -0.042 | -0.15, 0.07 | -0.024 | -0.15, 0.10 | 0.061 | -0.09, 0.21 |
| % living in crowded housing units | 0.195*** | 0.09, 0.30 | 0.103* | -0.01, 0.21 | 0.125** | 0.02, 0.23 | 0.107 | -0.02, 0.23 | 0.028 | -0.12, 0.18 |
| % public transportation for commute | -0.013 | -0.12, 0.09 | 0.022 | -0.09, 0.13 | -0.060 | -0.17, 0.05 | -0.020 | -0.15, 0.11 | -0.064 | -0.21, 0.09 |
| % with no vehicle | -0.202*** | -0.30, -0.10 | -0.190*** | -0.29, -0.08 | -0.191*** | -0.29, -0.08 | -0.106 | -0.23, 0.02 | -0.105 | -0.25, 0.04 |
| % vacant housing | 0.051 | -0.06, 0.16 | -0.007 | -0.12, 0.10 | -0.003 | -0.11, 0.11 | -0.016 | -0.14, 0.11 | 0.082 | -0.07, 0.23 |

* Bonferroni-adjusted p<0.05, ** Bonferroni-adjusted p<0.01, *** Bonferroni-adjusted p<0.001

# Appendix D. Sensitivity Analyses

Table D-1. Spearman correlations and Bonferroni-adjusted 95% CIs between median hospital-reported prices and social risk factors by price type, among HSAs with prices for ≥1 service reported.

|  | **Chargemaster Price (n=1,481)** | | **Cash Price (n=1,432)** | | **Commercial Price (n=1,417)** | | **Medicare Price (n=1,175)** | | **Medicaid Price (n=899)** | |
| --- | --- | --- | --- | --- | --- | --- | --- | --- | --- | --- |
|  | r_s_ | Bonferroni 95% CI | r_s_ | Bonferroni 95% CI | r_s_ | Bonferroni 95% CI | r_s_ | Bonferroni 95% CI | r_s_ | Bonferroni 95% CI |
| Total |  |  |  |  |  |  |  |  |  |  |
| **Socioeconomic position** | | | | | | | | | | |
| Median household income | 0.016 | -0.08, 0.12 | 0.050 | -0.05, 0.15 | 0.010 | -0.09, 0.11 | 0.076 | -0.04, 0.19 | -0.007 | -0.13, 0.12 |
| % uninsured | 0.064 | -0.04, 0.16 | 0.037 | -0.06, 0.14 | 0.053 | -0.05, 0.15 | 0.011 | -0.10, 0.12 | 0.003 | -0.12, 0.13 |
| % Medicare | -0.019 | -0.12, 0.08 | -0.044 | -0.14, 0.06 | -0.006 | -0.11, 0.10 | -0.055 | -0.17, 0.06 | 0.033 | -0.09, 0.16 |
| % Medicaid | -0.036 | -0.14, 0.06 | -0.063 | -0.16, 0.04 | -0.070 | -0.17, 0.03 | -0.081 | -0.19, 0.03 | -0.059 | -0.19, 0.07 |
| % with less than high school diploma | 0.009 | -0.09, 0.11 | -0.007 | -0.11, 0.09 | 0.019 | -0.08, 0.12 | -0.033 | -0.14, 0.08 | -0.001 | -0.13, 0.13 |
| % unemployed | 0.033 | -0.07, 0.13 | 0.035 | -0.07, 0.14 | -0.008 | -0.11, 0.09 | -0.021 | -0.13, 0.09 | -0.015 | -0.14, 0.11 |
| % receiving food stamps | -0.027 | -0.13, 0.07 | -0.061 | -0.16, 0.04 | -0.031 | -0.13, 0.07 | -0.065 | -0.18, 0.05 | -0.021 | -0.15, 0.11 |
| **Race, Ethnicity, and Cultural Context** | | | | | | | | | | |
| % Hispanic | 0.082 | -0.02, 0.18 | 0.121** | 0.02, 0.22 | 0.054 | -0.05, 0.16 | 0.057 | -0.06, 0.17 | -0.007 | -0.13, 0.12 |
| % NH Black | -0.011 | -0.11, 0.09 | 0.013 | -0.09, 0.11 | 0.011 | -0.09, 0.11 | 0.012 | -0.10, 0.12 | -0.030 | -0.16, 0.10 |
| % NH Other | 0.056 | -0.04, 0.15 | 0.077 | -0.02, 0.18 | 0.001 | -0.10, 0.10 | 0.063 | -0.05, 0.17 | -0.040 | -0.17, 0.09 |
| % limited English proficiency | 0.056 | -0.04, 0.16 | 0.116** | 0.02, 0.22 | 0.041 | -0.06, 0.14 | 0.058 | -0.05, 0.17 | -0.019 | -0.15, 0.11 |
| % non-citizens | 0.062 | -0.04, 0.16 | 0.114** | 0.01, 0.21 | 0.028 | -0.07, 0.13 | 0.062 | -0.05, 0.17 | -0.008 | -0.14, 0.12 |
| **Gender - % female** | 0.014 | -0.09, 0.11 | 0.028 | -0.07, 0.13 | 0.014 | -0.09, 0.12 | -0.048 | -0.16, 0.06 | -0.016 | -0.14, 0.11 |
| **Social Relationships** | | | | | | | | | | |
| % married | 0.041 | -0.06, 0.14 | 0.036 | -0.07, 0.14 | 0.061 | -0.04, 0.16 | 0.115* | 0.00, 0.22 | 0.080 | -0.05, 0.21 |
| % living alone | -0.016 | -0.12, 0.08 | -0.027 | -0.13, 0.07 | -0.029 | -0.13, 0.07 | -0.126** | -0.23, -0.02 | -0.001 | -0.13, 0.13 |
| % single parent families | 0.011 | -0.09, 0.11 | -0.025 | -0.13, 0.08 | -0.007 | -0.11, 0.10 | -0.035 | -0.15, 0.08 | -0.025 | -0.15, 0.10 |
| **Residential and Community Context** | | | | | | | | | | |
| Gini Index | 0.038 | -0.06, 0.14 | 0.026 | -0.08, 0.13 | -0.032 | -0.13, 0.07 | -0.070 | -0.18, 0.04 | -0.036 | -0.16, 0.09 |
| Social Deprivation Index | 0.000 | -0.10, 0.10 | -0.026 | -0.13, 0.08 | -0.038 | -0.14, 0.06 | -0.083 | -0.19, 0.03 | -0.056 | -0.18, 0.07 |
| % rural | -0.037 | -0.14, 0.06 | -0.093 | -0.19, 0.01 | -0.011 | -0.11, 0.09 | -0.045 | -0.16, 0.07 | 0.044 | -0.08, 0.17 |
| % living in crowded housing units | 0.053 | -0.05, 0.15 | 0.064 | -0.04, 0.16 | 0.007 | -0.10, 0.11 | 0.030 | -0.08, 0.14 | -0.031 | -0.16, 0.10 |
| % public transportation for commute | -0.001 | -0.10, 0.10 | 0.043 | -0.06, 0.14 | -0.030 | -0.13, 0.07 | -0.030 | -0.14, 0.08 | -0.063 | -0.19, 0.07 |
| % with no vehicle | -0.080 | -0.18, 0.02 | -0.068 | -0.17, 0.03 | -0.055 | -0.16, 0.05 | -0.136*** | -0.24, -0.02 | -0.049 | -0.18, 0.08 |
| % vacant housing | -0.018 | -0.12, 0.08 | -0.026 | -0.13, 0.08 | -0.057 | -0.16, 0.05 | -0.076 | -0.19, 0.04 | -0.032 | -0.16, 0.10 |

* Bonferroni-adjusted p<0.05, ** Bonferroni-adjusted p<0.01, *** Bonferroni-adjusted p<0.001

Table D-2. Spearman correlations and Bonferroni-adjusted 95% CIs between median hospital-reported prices and social risk factors by price type, among HRRs with prices for ≥8 services reported.

|  | **Chargemaster Price (n=294)** | | **Cash Price (n=288)** | | **Commercial Price (n=290)** | | **Medicare Price (n=264)** | | **Medicaid Price (n=228)** | | |  |
| --- | --- | --- | --- | --- | --- | --- | --- | --- | --- | --- | --- | --- |
|  | r_s_ | Bonferroni 95% CI | r_s_ | Bonferroni 95% CI | r_s_ | Bonferroni 95% CI | r_s_ | Bonferroni 95% CI | | r_s_ | Bonferroni 95% CI | |
| **Socioeconomic position** | | | | | | | | | | | |  |
| Median household income | 0.011 | -0.21, 0.23 | 0.119 | -0.11, 0.33 | 0.059 | -0.17, 0.28 | -0.023 | -0.25, 0.21 | | -0.036 | -0.28, 0.22 | |
| % uninsured | 0.292*** | 0.08, 0.48 | 0.153 | -0.07, 0.36 | 0.254** | 0.03, 0.45 | 0.151 | -0.09, 0.37 | | 0.168 | -0.09, 0.40 | |
| % Medicare | -0.116 | -0.33, 0.11 | -0.158 | -0.37, 0.07 | -0.131 | -0.34, 0.09 | -0.081 | -0.31, 0.16 | | 0.004 | -0.25, 0.25 | |
| % Medicaid | 0.008 | -0.21, 0.23 | -0.121 | -0.34, 0.11 | -0.189 | -0.39, 0.04 | -0.041 | -0.27, 0.19 | | -0.116 | -0.36, 0.14 | |
| % with less than high school diploma | 0.125 | -0.10, 0.34 | -0.058 | -0.28, 0.17 | -0.003 | -0.23, 0.22 | -0.024 | -0.26, 0.21 | | -0.011 | -0.26, 0.24 | |
| % unemployed | 0.165 | -0.06, 0.37 | 0.032 | -0.19, 0.25 | 0.025 | -0.20, 0.25 | 0.038 | -0.20, 0.27 | | -0.052 | -0.30, 0.20 | |
| % receiving food stamps | 0.009 | -0.21, 0.23 | -0.138 | -0.35, 0.09 | -0.121 | -0.34, 0.10 | -0.005 | -0.24, 0.23 | | -0.038 | -0.29, 0.21 | |
| **Race, Ethnicity, and Cultural Context** | | | | | | | | | | | |  |
| % Hispanic | 0.437*** | 0.24, 0.60 | 0.393*** | 0.19, 0.57 | 0.282*** | 0.06, 0.47 | 0.137 | -0.10, 0.36 | | 0.037 | -0.22, 0.28 | |
| % NH Black | 0.106 | -0.12, 0.32 | -0.051 | -0.27, 0.17 | 0.121 | -0.10, 0.33 | 0.025 | -0.21, 0.26 | | 0.106 | -0.15, 0.35 | |
| % NH Other | 0.031 | -0.19, 0.25 | 0.093 | -0.13, 0.31 | 0.112 | -0.11, 0.33 | 0.107 | -0.13, 0.33 | | -0.044 | -0.29, 0.21 | |
| % limited English proficiency | 0.352*** | 0.14, 0.53 | 0.295*** | 0.08, 0.49 | 0.218* | 0.00, 0.42 | 0.059 | -0.18, 0.29 | | 0.023 | -0.23, 0.27 | |
| % non-citizens | 0.321*** | 0.11, 0.51 | 0.286*** | 0.07, 0.48 | 0.242** | 0.02, 0.44 | 0.074 | -0.16, 0.30 | | 0.064 | -0.19, 0.31 | |
| **Gender - % female** | -0.017 | -0.24, 0.20 | -0.180 | -0.39, 0.05 | -0.034 | -0.26, 0.19 | -0.131 | -0.35, 0.11 | | 0.058 | -0.20, 0.30 | |
| **Social Relationships** | | | | | | | | | | | |  |
| % married | -0.135 | -0.35, 0.09 | 0.000 | -0.22, 0.22 | -0.046 | -0.27, 0.18 | -0.010 | -0.24, 0.22 | | -0.027 | -0.28, 0.23 | |
| % living alone | -0.241** | -0.44, -0.02 | -0.251** | -0.45, -0.03 | -0.131 | -0.34, 0.09 | -0.067 | -0.30, 0.17 | | 0.023 | -0.23, 0.27 | |
| % single parent families | 0.166 | -0.06, 0.37 | 0.015 | -0.21, 0.24 | 0.075 | -0.15, 0.29 | 0.053 | -0.18, 0.28 | | 0.058 | -0.20, 0.30 | |
| **Residential and Community Context** | | | | | | | | | | | |  |
| Gini Index | 0.051 | -0.17, 0.27 | -0.086 | -0.30, 0.14 | -0.001 | -0.22, 0.22 | 0.090 | -0.15, 0.32 | | 0.135 | -0.12, 0.37 | |
| Social Deprivation Index | 0.141 | -0.08, 0.35 | -0.040 | -0.26, 0.18 | 0.016 | -0.21, 0.24 | 0.034 | -0.20, 0.27 | | 0.011 | -0.24, 0.26 | |
| % rural | -0.240** | -0.44, -0.02 | -0.205 | -0.41, 0.02 | -0.187 | -0.39, 0.04 | -0.063 | -0.29, 0.17 | | 0.010 | -0.24, 0.26 | |
| % living in crowded housing units | 0.264** | 0.05, 0.46 | 0.213* | -0.01, 0.42 | 0.187 | -0.04, 0.39 | 0.148 | -0.09, 0.37 | | -0.006 | -0.26, 0.24 | |
| % public transportation for commute | 0.060 | -0.16, 0.28 | 0.128 | -0.10, 0.34 | 0.047 | -0.18, 0.27 | -0.031 | -0.26, 0.20 | | -0.031 | -0.28, 0.22 | |
| % with no vehicle | -0.245** | -0.44, -0.02 | -0.278*** | -0.47, -0.06 | -0.253** | -0.45, -0.03 | -0.134 | -0.36, 0.10 | | -0.065 | -0.31, 0.19 | |
| % vacant housing | 0.089 | -0.14, 0.30 | -0.006 | -0.23, 0.22 | 0.056 | -0.17, 0.28 | 0.066 | -0.17, 0.29 | | 0.196 | -0.06, 0.43 | |

* Bonferroni-adjusted p<0.05, ** Bonferroni-adjusted p<0.01, *** Bonferroni-adjusted p<0.001
